# Analysis of adult disease characteristics and mortality on MIMIC-III

**DOI:** 10.1371/journal.pone.0232176

**Published:** 2020-04-30

**Authors:** Zheng Dai, Siru Liu, Jinfa Wu, Mengdie Li, Jialin Liu, Ke Li

**Affiliations:** 1 School of Life Science & Technology, University of Electronic Science & Technology of China, Chengdu, China; 2 Department of Biomedical Informatics, University of Utah, Salt Lake City, Utah, United States of America; 3 Department of Medical Informatics, West China Medical School, Sichuan University, Chengdu, China; 4 Information Center, West China Hospital, Sichuan University, Chengdu, China; Karolinska Institutet, SWEDEN

## Abstract

**Purpose:**

To deeply analyze the basic information and disease information of adult patients in the MIMIC-III (Medical Information Mart for Intensive Care III) database, and provide data reference for clinicians and researchers.

**Materials and methods:**

Tableau2019.1.0 and Navicat12.0.29 were used for data analysis and extraction of disease distribution of adult patients in the MIMIC-III database.

**Result:**

A total of 38,163 adult patients were included in the MIMIC-III database. Only 38,156 patients with the first diagnosis were selected. Among them, 21,598 were males accounting for 56.6% the median age was 66 years (Q1-Q3: 53–78), the median length of a hospital stay was 7 days (Q1-Q3: 4–12), and the median length of an ICU stay was 2.1 days (Q1-Q3: 1.2–4.1). Septicemia was the disease with the highest mortality rate among patients and the total mortality rate was 48.9%. The disease with the largest number of patients at the last time was other forms of chronic ischemic heart disease.

**Conclusion:**

By analyzing the patients’ basic information, the admission spectrum and the disease morbidity and mortality can help more researchers understand the MIMIC-III database and facilitate further research.

## 1. Introduction

In order to understand the nature of the disease, many studies have been carried out to determine the pathogenesis, course, and complications of the disease [1–3]. MIMIC-III (Medical Information Mart for Intensive Care III) is a large, freely-available database comprising de-identified health-related data associated with over forty thousand patients who stayed in critical care units of the Beth Israel Deaconess Medical Center between 2001 and 2012 [4]. The data in the MIMIC-III database is more reliable and complete than the data in the MIMIC-II database. Many researchers have conducted a lot of research on the MIMIC-III database. Kurniati A P [5] used process mining to study oncology. Mengling F’s [6] study was designed to examine the association of transthoracic echocardiography with 28-day mortality specifically in patients who suffered from sepsis. Parreco J P [7] used supervised machine learning to predict central line-associated bloodstream infection (CLABSI). Some scholars have used deep learning [8,9] and visualization methods [10,11] to study the MIMIC-III database and get better results. There are also many other researchers [12–15] using other methods to study the MIMIC-III database.

Recent researches have leveraged machine learning techniques to predict the mortality rate of various diseases. In order to solve clinical problems in a meaningful way, researchers need to understand clinicians’ needs. Because clinicians lack knowledge of SQL programming and EHR database programming [16–18], the clinical questions that they raised cannot be supported by data. Therefore, in order to promote the communication and cooperation between clinicians and researchers [18], a detailed analysis of disease information based on the MIMIC-III database was conducted in this paper to provide data reference for researchers.

## 2. Materials and methods

### 2.1. Data source

This study used the publicly available Multiparameter Intelligent Monitoring in Intensive Care (MIMIC) III database version 1.4 [4]. The MIMIC-III database is derived from the private medical record of the ICU of the Boston Medical Center in the United States. All patients’ data were anonymized prior to extraction and data analysis. The creation, maintenance, and use of the MIMIC-III database were approved by the institutional review boards of the Massachusetts Institute of Technology and Beth Israel Deaconess Medical Center [19]. The database contains detailed information on patients’ vital signs, laboratory tests, and diagnosis codes for research by global scholars. The MIMIC-III database contains 46,520 patients at the unique patient level, of whom 38,163 are adult patients, 38,161 patients were last admitted to the ICU, and 38,156 patients have the first diagnosis.

### 2.2. Data selection

Because juvenile patients have different physical status from adult patients, this article only selected adult patients to conduct data analysis. The research in this paper is based on unique patient levels. All data were obtained at a unique patient level. Because a patient may have multiple admission records, we selected the last admission record. In the last admission record, we selected the last ICU record for statistical analysis. 38,161 patients were last admitted to the ICU. In this study, only the first diagnosis was selected as the object of data analysis, and the disease classification was gradually refined according to the primary International Classification of Diseases (ICD-9) codes [20]. Because the MIMIC-III database only includes the patients’ admission time and discharge time, we used the discharge time minus the admission time to obtain the patients’ hospitalization days. The date of death of each patient was recorded in the MIMIC-III database. We used the date of death of each patient minus the date of discharge to obtain how many days the patients died after being discharged. Then we selected patients who died within 90 days for analysis.

### 2.3. Research methods

The research in this article was divided into two parts the first part was the statistics and analysis of basic information of all patients in MIMIC-III database, and the second part was the distribution of diseases. The first part describes the patients’ age, gender, ethnicity, admission time and distribution of ICU types. We used SQL programming in Navicat12.0.29 to extract the required indicator data from the database and then imported the extracted data into Tableau2019.1.0. We used table query and calculated fields in Tableau2019.1.0 to filter unique data and then performed the visual analysis to get the final result.

In the second part, we analyzed the basic information of ten diseases with the largest number of patients, and the mortality and the ICU distribution of the ten diseases. Firstly, according to the ICD-9 coding standard, the number of patients and deaths of each disease were counted, and the ten diseases with the largest number of patients and the ten diseases with the highest mortality were screened. Then, we counted the basic information of patients for each disease. According to the analysis methods mentioned in the first part of this section, the ICU distribution of the ten diseases with the largest number of patients was made to reflect the main diseases of each ICU.

## 3. Result

### 3.1. Basic information of all patients in MIMIC-III

We divided patients into three parts: death in hospital, 90-day death after discharge, and no death within 90-day after discharge. We divided patients into three categories and gained their information separately, so we can understand the disease characteristics of different populations more clearly.

We analyzed such as length of stay, age, gender. Age and length of stay are expressed in median (inter-quartile range). Two of the 90-day-dead patients who were discharged from the hospital had incorrect information that the time of death was greater than the time of admission. Therefore, the basic information of the two patients was not counted in the statistics. It can be seen from the Table 1 that the dead patients (include death in the hospital and 90-day death after discharge) are older, 74 years (Q1-Q3: 60–83) and 76 years (Q1-Q3: 64–84), while the non-dead patients are relatively young, 64 years (Q1-Q3: 51–76). The mortality of patients in the MIMIC-III database is 23.2%. Data analysis showed that older people were more likely to be admitted to the ICU and had a higher risk of death. We should pay more attention to the physiological indicators of patients above the median age of 74 years old to improve their survival rate.

**Table 1:** Patients’ basic information in MIMIC-III.

Type	Age, years, median (Q1-Q3)	Male (%)	Days in hospital, median days (Q1-Q3)	Days in ICU, median days (Q1-Q3)	Number of patients (%)
Death in the hospital	74 (60–83)	3046 (54.5%)	6 (2–13)	3.1 (1.3–7.5)	5697 (15.0%)
90-day of death after discharge	76 (64–84)	1690 (53.8%)	9 (5–16)	2.7 (1.5–5.3)	3140+2(8.2%)
No death within 90-day after discharge	64 (51–76)	16862 (57.5%)	7 (4–11)	2.0 (1.2–3.5)	29324(76.8%)
Sum	66 (53–78)	21598 (56.6%)	7 (4–12)	2.1 (1.2–4.1)	38163(100%)

**Fig 1:**
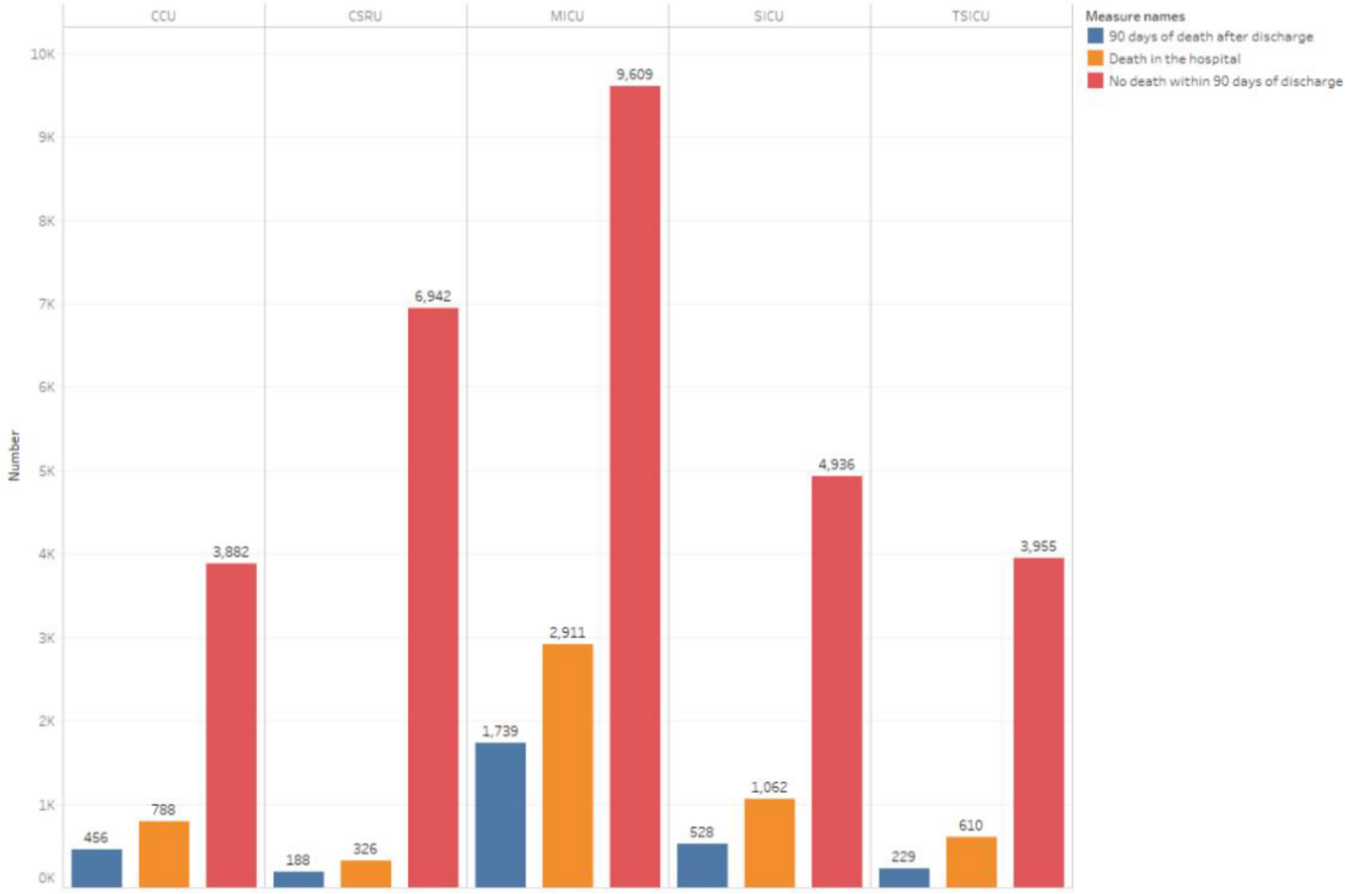
ICU distribution of all patients admitted to the ICU (Total number of patients is 38161).

**Fig 2:**
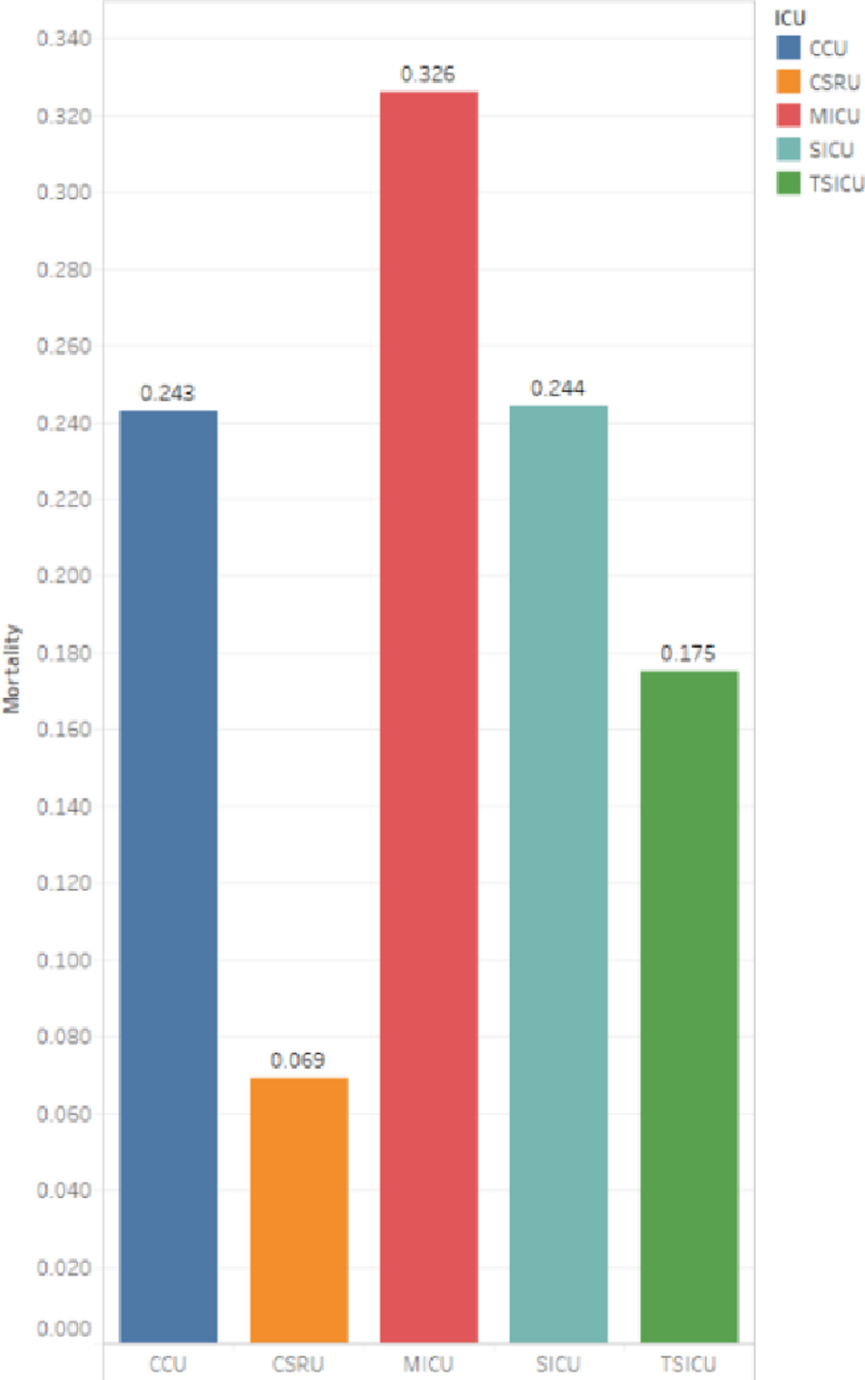
ICU mortality of all patients (in-hospital + 90-day after discharge).

We counted the distribution of patients in different ICUs (Fig 1). The ICU with the largest number of patients is the Medical Intensive Care Unit (MICU), and the number of patients who died in the hospital and the number of patients non-dead within 90-day after discharge in the MICU are also much higher than those in other ICUs. It shows that the internal disease is the disease with the largest number of patients and the highest mortality rate. In subsequent studies, we can study the physiological indicators of patients in MICU and use machine learning to predict the health of patients. Fig 1 also shows that the number of patients admitted to Cardiac Surgery Intensive Care Unit (CSRU) was second only to MICU, but the number of deaths in hospital and the number of 90-day deaths after discharge is the lowest compared to other ICUs. In Fig 2, we counted the mortality of patients admitted to each type of ICU, and we found that the highest mortality rate was 32.6% in MICU. Mortality exceeds 24% in both CCU (24.3%) and SICU (24.4%).

**Fig 3:**
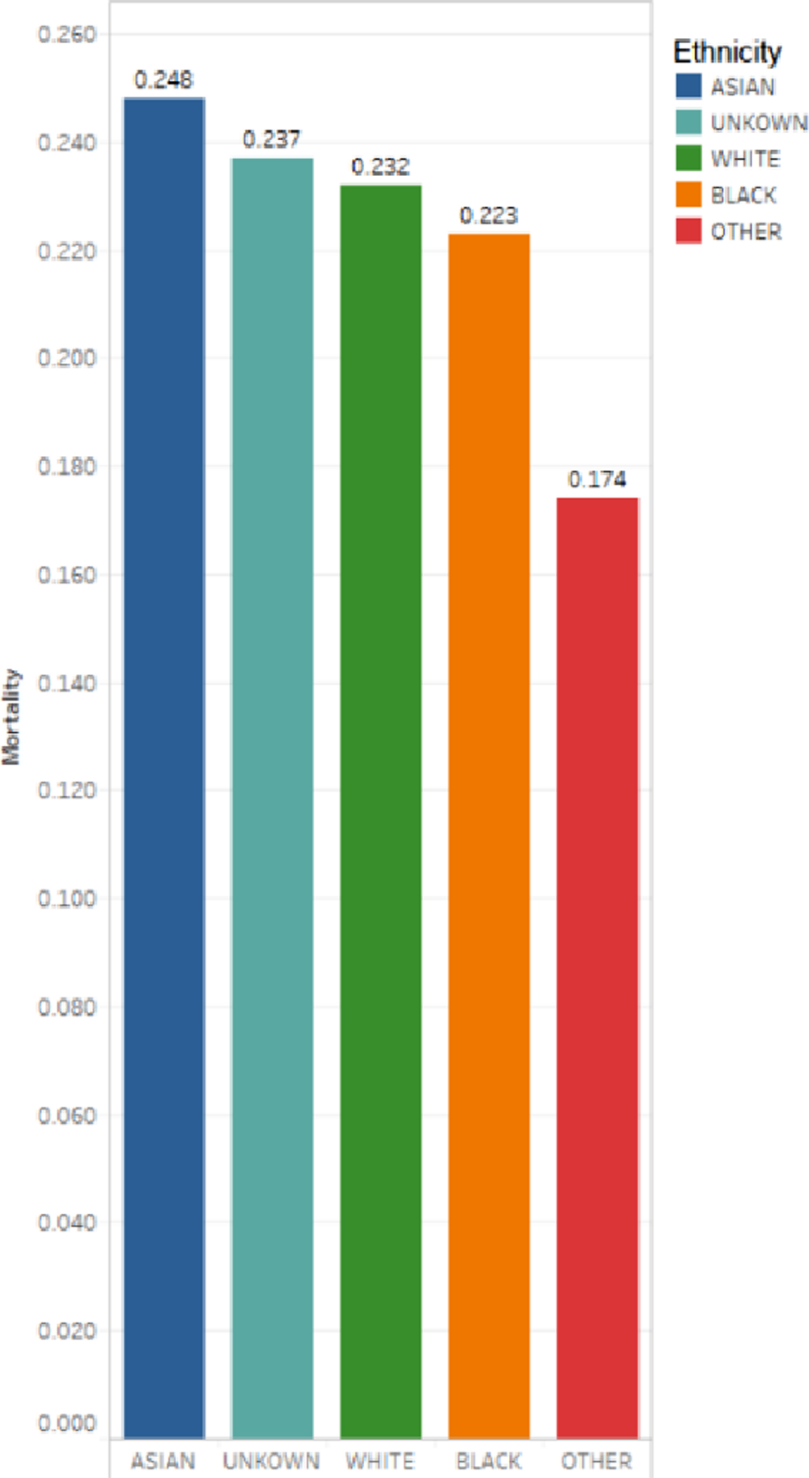
Mortality of different race (It is calculated as: The number of deaths per race/the total number of patients per race).

**Fig 4:**
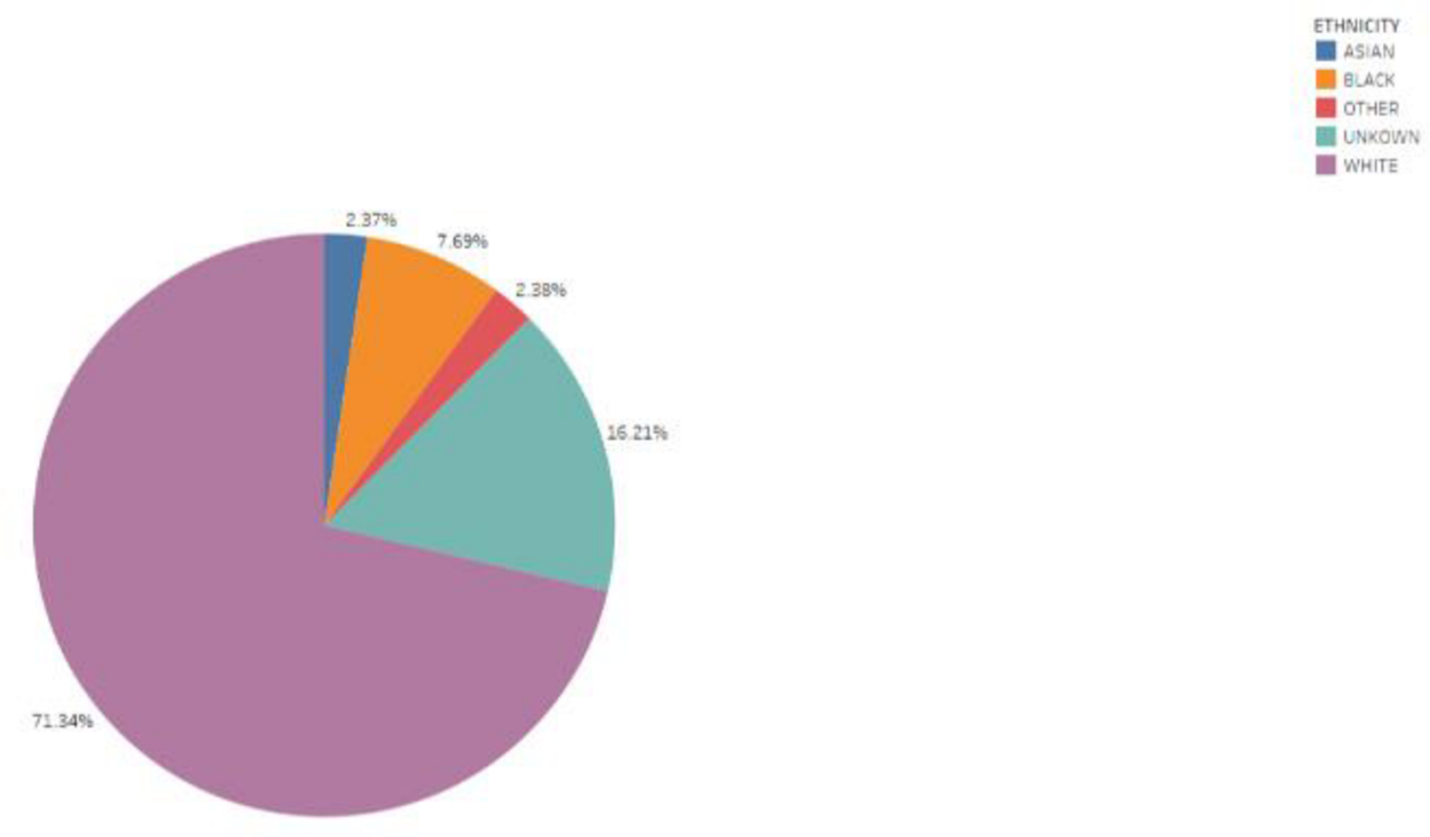
Ethnic distribution of all patients (Total number of patients is 38161).

**Fig 5:**
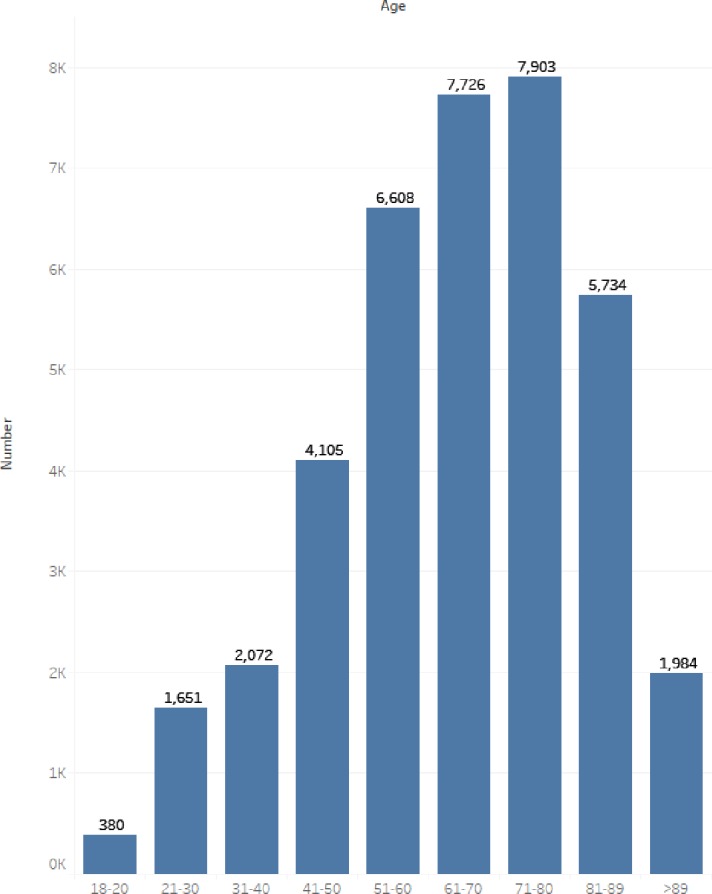
Age distribution of all admitted patients (Total number of patients is 38163).

We analyzed the ethnic mortality of patients (Fig 3). We find Asians have the highest mortality rate, at 24.8%. Fig 4 depicts a pie chart of the ethnic distribution of all adult patients in the MIMIC-III database. The ethnic distribution and proportion can be clearly seen. Because the database does not completely record the race of patients, many patients only say their nationality, so it is not possible to specify which race. Therefore, “UNKOWN” and “OTHER” exist in the ethnic classification. In Fig 5 we have calculated the age distribution of patients in MIMIC-III. On the whole, we can find that ICU patients are mainly elderly people. Specifically, we can see that the number of patients in the 71–80 age group is the largest, followed by the 61–70 age group. Therefore, we think that the elderly at these ages have a high risk of diseases, and it is particularly important to protect the elderly in their daily lives.

### 3.2. The distribution of diseases in MIMIC-III

According to the coding standard of ICD-9, this article counted the top 10 diseases with the largest number of patients admitted to the hospital according to the first diagnostic criteria (Table 2). A total of 38,163 patients were admitted, and 38,156 patients had the first diagnosis. The total number of the top 10 diseases with the largest number of patients is 15020. Because we only selected the patients’ first diagnosis as the data analysis, it is equivalent to each patient suffering from only one disease. No cross-replicated data for the diseases. The disease information of patients was counted according to the first diagnosis. The most common disease of patients admitted to hospital is Other forms of chronic ischemic heart disease, but the mortality rate of this category is the lowest in the ten diseases. The 90-day mortality rate and in-hospital mortality of Septicemia disease are the highest among the ten diseases. Heart failure has the highest median age among the ten diseases it is 77 years (Q1-Q3:66–85).

**Table 2:** Basic information of ten diseases with the largest number of patients.

No.	Disease/ICD-9	Age, years, median (Q1-Q3)	Male (%)	Days in hospital, median days (Q1-Q3)	Days in ICU, median days (Q1-Q3)	Death in the hospital (%)	90 days of death after discharge (%)	Number of patients
1	Other forms of chronic ischemic heart disease /414	68(60–75)	2371(77.3%)	6(5–9)	2.0(1.2–3.1)	34(1.1%)	47(1.5%)	3069
2	Acute myocardial infarction/410	70(59–81)	1723(64.1%)	6(3–10)	2.1(1.2–3.9)	305(11.3%)	173(6.4%)	2689
3	Septicemia/038	72(59–83)	1358(52.5%)	7(4–13)	2.9(1.6–6.5)	952(36.8%)	314(12.1%)	2587
4	Other diseases of endocardium /424	71(60–79)	865(59.4%)	7(5–10)	2.1(1.2–3.3)	24(1.6%)	28(1.9%)	1456
5	Other diseases of lung/518	69(56–80)	544(51.0%)	8(4–15)	4.7(2.1–9.8)	331(31.0%)	144(13.5%)	1067
6	Heart failure/428	77(66–85)	533(56.2%)	8(5–13)	2.7(1.4–5.1)	182(19.2%)	153(16.1%)	948
7	Intracerebral hemorrhage/431	72(60–82)	491(52.9%)	6(3–12)	2.1(1.1–5.1)	334(36.0%)	83(8.9%)	929
8	Subarachnoid, subdural, and extradural hemorrhage, following injury/852	76(58–85)	484(54.8%)	5(3–10)	1.8(1.0–3.4)	164(18.6%)	69(7.8%)	883
9	Cardiac dysrhythmias /427	70(59–81)	463(60.6%)	5(2–8)	1.9(1.1–3.4)	125(16.4%)	56(7.3%)	764
10	Occlusion of cerebral arteries/434	76(63–85)	292(46.5%)	6(4–11)	2.1(1.2–3.9)	158(25.2%)	75(11.9%)	628

**Fig 6:**
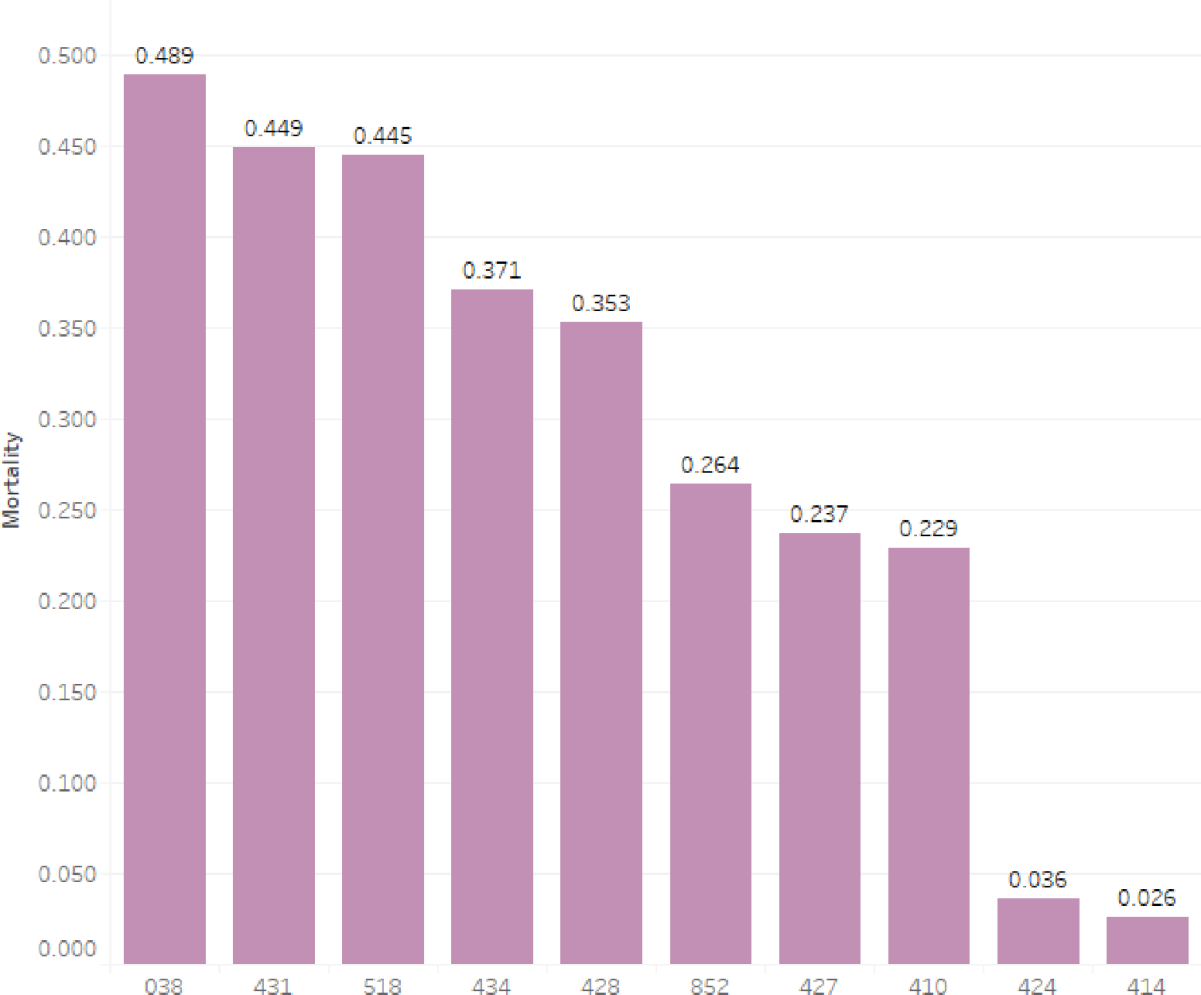
Mortality of ten diseases with the largest number of patients (all dead patients: death in-hospital + 90-day of death after discharge).

Fig 6 shows the mortality rate of the top 10 diseases with the largest number of patients. The disease with the highest mortality rate is Septicemia, and the mortality rate is 48.9%. Followed by Intracerebral hemorrhage and Other diseases of lung, the mortality rate of which also exceeded 44%. Patients with Other diseases of lung have the longest ICU stay. The days reached 4.68 (Q1-Q3: 2.1–9.8).

**Fig 7:**
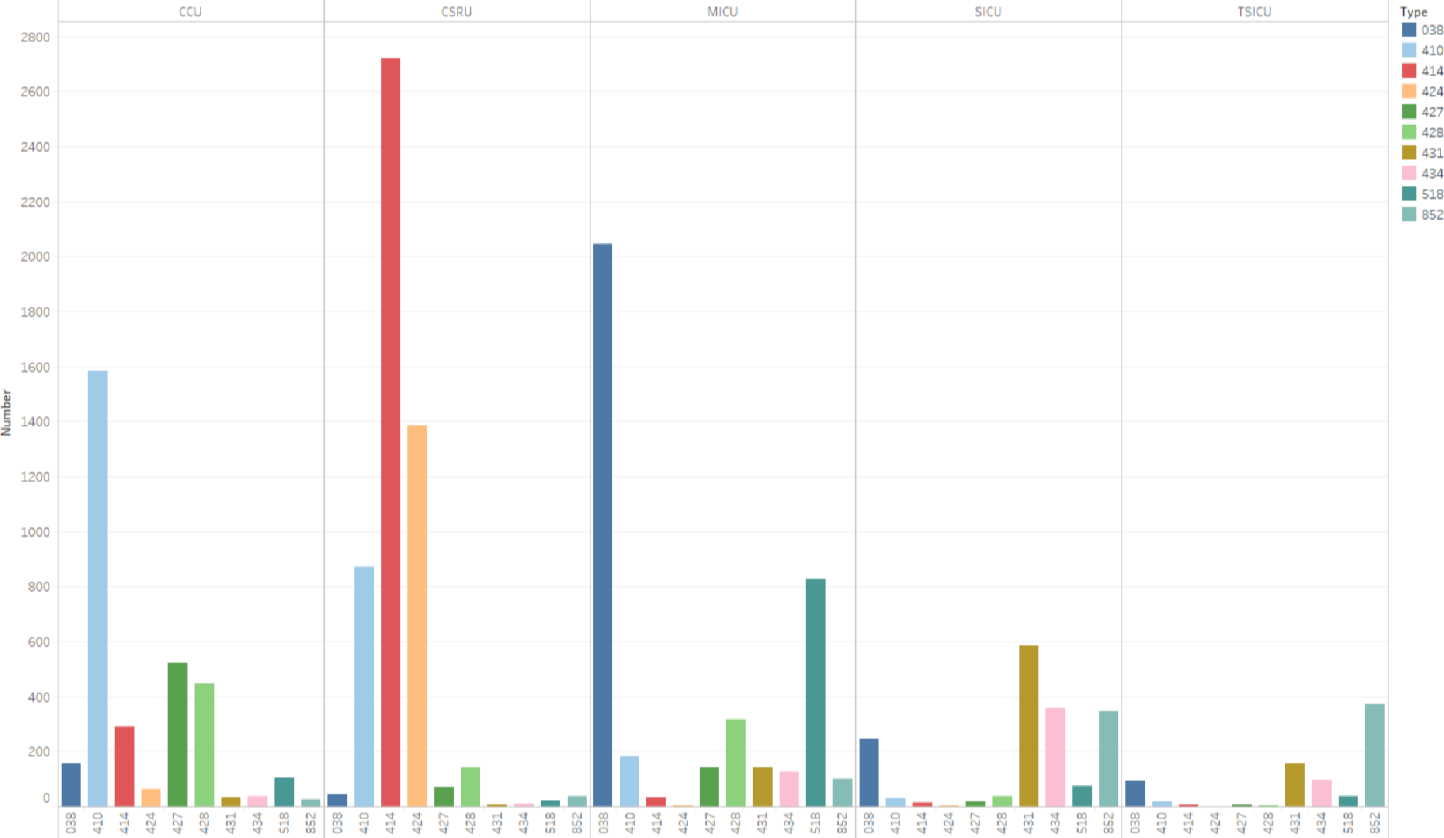
The distribution of ten diseases with the largest number of patients in ICU.

Fig 7 shows the distribution of ten diseases with the largest number of patients in ICU. The disease with the greatest number of patients is Other forms of chronic ischemic heart disease in the five ICUs. Followed by Other diseases of endocardium and Acute myocardial infarction. In the CCU, the disease with the greatest number of patients is Acute myocardial infarction. In the MICU, the disease with the greatest number of patients is Septicemia. In SICU and TSICU, the number of patients is relatively small. The main diseases are Intracerebral hemorrhage and Subarachnoid, subdural, and extradural hemorrhage, followed by injury and Occlusion of cerebral arteries.

## 4. Discussion

The MIMIC-III database is the first large ICU database that is open to the public free of charge. It has a really large dataset with rich types of medical data, providing high-quality data resources for clinical research, mining and building a knowledge base. This study was based on the latest version of the MIMIC-III database to analyze the information of diseases and the basic information of adult patients who had entered the ICU for the last time.

The research in this paper was mainly divided into two parts. In the first part, we divided the admission patients into three categories: death in the hospital, 90-day of death after discharge, and no death within 90-day after discharge. Referring to some scholars’ research [21,22], this paper divided patients into three categories, which can better understand the disease characteristics of patients in the MIMIC database. The second part was to introduce the basic information about the top 10 diseases with the largest number of patients, including the length of hospital stay, gender, age, in-hospital mortality, and 90-day mortality. Based on the disease information of the admitted patients, the mortality of each disease and the distribution of ICUs were calculated.

Fan [18] also made a statistical analysis of the MIMIC-III database. The distribution of disease spectrum in patients of the same age is described in detail. However, the whole study basically analyzed the patients as a whole and does not classify the patients. It is not clear about the changes in patients at different stages. Therefore, we divided the patients into three parts for the analysis of related indicators and mortality. This can help us understand the patients’ situation more clearly. And they selected the patients who were admitted for the first time. In our study, we selected the patients who were admitted to the hospital for the last time and they were more representative. Their research is based on the human body systems. There is no analysis of specific diseases. In our study, the basic information and mortality of the top 10 diseases with the largest number of patients were analyzed. We also calculated the prevalence of the top 10 diseases with the largest number of patients in different ICUs, so that we can see the types of the main diseases in each ICU more clearly.

This study was subject to some limitations: First, we extracted data from the MIMIC-III database, which is a retrospective study with selective bias. Second, this study mainly involved Caucasian, African American and Asians are very few. Due to ethnic differences, the results of this study need to be further verified in other ethnic groups. Third, we did not conduct influencing factors analysis and predictive analysis for these diseases. Influencing factors analysis and predictive analysis can better remind people how to prevent the diseases from the onset, predict the progression of the diseases according to the patients’ condition and basic physiological characteristics, and provide patients with better prognosis and rational allocation of medical resources. This will be the main direction of our next research.

## 5. Conclusion

In summary, this study provides a detailed analysis and description of the case records and disease distribution of adult patients entering the ICU for the MIMIC-III database. It provides the statistics of patients’ basic information and the top 10 diseases with the largest number of patients and the distribution of the ten diseases. This paper implements a complete data analysis process for the MIMIC-III database, and systematically paves the way for clinicians and researchers to conduct data research in the medical field.

## Supporting information

S1 Data (DOCX)

S1 Checklist *PLOS ONE* clinical studies checklist. (DOCX)
