# Supplementary material for: Analysis of adult disease characteristics and mortality on MIMIC-III
Source: PLoS One. 2020 Apr 30;15(4):e0232176. doi: 10.1371/journal.pone.0232176 (PMC7192440; doi:10.1371/journal.pone.0232176)
Supplement: S1 Data — (DOCX) [file pone.0232176.s001.docx]

MIMIC-III v1.4

a) A description of the data set and the third-party source

MIMIC-III (Medical Information Mart for Intensive Care) is a large, single-center database comprising information relating to patients admitted to critical care units at a large tertiary care hospital. Data includes vital signs, medications, laboratory measurements, observations and notes charted by care providers, fluid balance, procedure codes, diagnostic codes, imaging reports, hospital length of stay, survival data, and more. The database supports applications including academic and industrial research, quality improvement initiatives, and higher education coursework.

The latest version of MIMIC is MIMIC-III v1.4, which comprises 61,532 intensive care unit stays: 53,432 stays for adult patients and 8,100 for neonatal patients. The data spans June 2001 - October 2012. The database, although de-identified, still contains detailed information regarding the clinical care of patients, so must be treated with appropriate care and respect.

Details are shown on MIMIC-III 's official website (<https://mimic.mit.edu/about/mimic/>)

b) If applicable, verification of permission to use the data set

The website does not provide the formal verification file. But we have registered as PhysioNet credentialed users to have permission to use the dataset.

c) All necessary contact information and/or URLs and accession codes that others would need to gain access to the data

**Complete the required training course**

Prior to requesting access to MIMIC, you will need to complete the CITI “Data or Specimens Only Research” course:

First register on the CITI program website, selecting “Massachusetts Institute of Technology Affiliates” as your affiliation (not “independent learner”): <https://www.citiprogram.org/index.cfm?pageID=154&icat=0&ac=0>

Follow the links to add a Massachusetts Institute of Technology Affiliates course. In the Human Subjects training category, select the “Data or Specimens Only Research” course

Complete the course and save a copy of your completion report. The completion report lists all modules completed, with dates and scores.

**Request access to MIMIC-III:**

Register for an account on PhysioNet: <https://physionet.org> . If you already have a PhysioNet account, log in.

Go to the project page at: <https://physionet.org/content/mimiciii/>.

Find the “Files” section in the project description.

Click “credentialed user” link, then follow the instructions to submit your application for credentialed access. Remember to provide your CITI completion report.

When your application has been approved you will receive emails containing instructions for downloading the database from PhysioNetWorks. Approval may take several business days, and will be delayed if your request is missing any required information.

Please be sure to provide all requested information. Submissions that are clearly incomplete, incorrect, or frivolous may be discarded without notice.

If you are a student or a postdoc, you must provide your supervisor’s name and contact information in the “reference” section of the form. If you are not listed in a directory or other easy-to-find page of your organization’s website, please provide the name and contact information of a reference such as a supervisor or colleague. Do not list yourself as reference.

Be sure to upload the completion report from the CITI “Data or Specimens Only Research” training program (PDF or image file). The completion report lists all modules completed, with dates and scores. If you would like to submit multiple pages, please combine them into a single pdf file.

**Downloading the MIMIC-III database**

PhysioNet

Once your application to access MIMIC has been approved, you will be granted access to the ‘MIMIC-III Clinical Database’ project page on PhysioNet:

<https://physionet.org/content/mimiciii/>

Data and build scripts

MIMIC is provided as a collection of comma-separated (CSV) files, along with scripts to help users import the data into popular database systems. Scripts are available in the MIMIC code repository for Postgres, MySQL, and Oracle:

<https://github.com/MIT-LCP/mimic-code/tree/master/buildmimic>

If you are familiar with Make, then you can directly clone the above repository and run make in order to build MIMIC on a PostgreSQL instance.

**Tutorials for installing MIMIC in a local Postgres database:**

<https://mimic.mit.edu/tutorials/>

d) Please confirm that the authors of the present study had no special access privileges in accessing data from MIMIC which other interested researchers would not have.

I confirm that authors in our group have the same access like other researchers.
